# A rare case of xanthogranuloma of the stomach masquerading as an advanced stage tumor

**DOI:** 10.1186/1477-7819-9-67

**Published:** 2011-07-02

**Authors:** Hiroyuki Kinoshita, Shunsuke Yamaguchi, Yoshifumi Sakata, Kazuo Arii, Kazunari Mori, Rieko Kodama

**Affiliations:** 1 Department of Surgery, Naga Municipal Hospital, 1282, Uchita, Kinokawa, Wakayama 649-6414, Japan; 2 Department of Pathology, Naga Municipal Hospital, Japan

**Keywords:** xanthogranuloma, early gastric cancer

## Abstract

**Background:**

Xanthogranuloma of the stomach is an extremely rare disease, and this lesion has only been found to coexist with early gastric cancer in 2 cases in the literature.

**Case presentation:**

We report a case of xanthogranuloma of the stomach combined with early gastric cancer that mimicked an advanced stage tumor. A 65-year-old female was referred to our hospital because of epigastralgia. During a physical examination, a defined abdominal mass was palpable in the region of the left hypochondrium. Imaging studies revealed an advanced gastric cancer, which was suspected of having infiltrated the abdominal wall. Total gastrectomy and resection of the regional lymph node and abdominal wall were performed. Histopathologic examination of the resected specimen demonstrated xanthogranuloma combined with early gastric cancer.

**Conclusion:**

Xanthogranuloma presenting as a form of SMT (submucosal tumor) of the stomach is an extremely rare disease, and diagnosing it preoperatively is difficult. Further accumulation and investigation of this entity is necessary.

## Background

Xanthogranuloma was first described by Oberling in 1935 [1]. Although it is known to develop in the gall bladder as xanthogranulomatous cholecystitis, xanthogranuloma of the stomach is an extremely rare disease, and only a few cases have been reported. Hence, we report a case of xanthogranuloma combined with early gastric cancer that mimicked an advanced stage tumor.

## Case report

**Figure 1:**
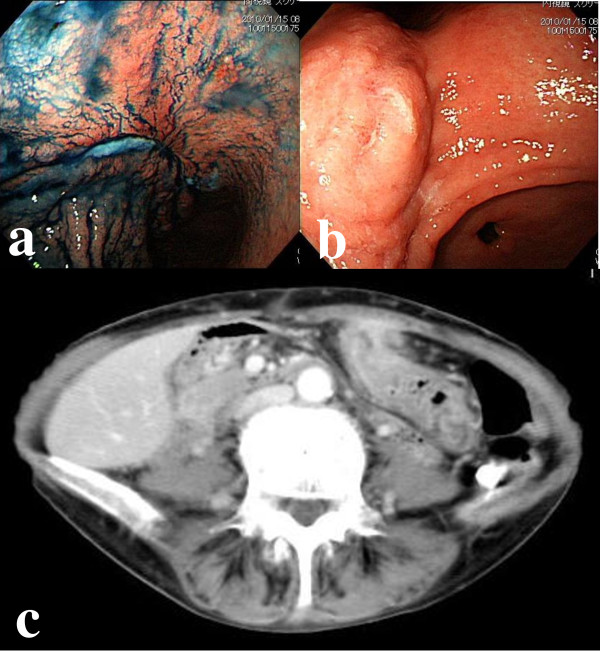
**Gastrointestinal endoscopic examination and Computed tomography**. a. A gastrointestinal endoscopic examination was performed and disclosed an ulcerated lesion in the lesser curvature of the gastric corpus located at 7 cm from the esophagogastric junction, which squashed and isolated the gastric folds from the rest of the stomach. b. An elevated lesion that appeared to be a submucosal tumor (SMT), which was suspected of being an advanced gastric cancer, was detected on the anal side of the ulcerated lesion. c. Computed tomography (CT) revealed thickening of the gastric wall and findings indicative of abdominal wall invasion.

A 65-year-old female was referred to Naga Municipal Hospital because of epigastralgia. During a physical examination, a defined abdominal mass was palpable in the region of the left hypochondrium. Neither anemia nor jaundice was present. Blood analysis showed a white blood cell count of 12.25 × 10^3^/μl. Her tumor marker serum levels were within the normal limits (carcinoembryonic antigen (CEA): 1.3 ng/ml, carbohydrate antigen (CA) 19-9: 10.1 U/ml). A gastrointestinal endoscopic examination was performed and disclosed an ulcerated lesion in the lesser curvature of the gastric corpus at about 7 cm from esophagogastric junction, which squashed and isolated the gastric folds from the rest of the stomach (Figure 1a), and an elevated lesion similar to a submucosal tumor (SMT), which was suspected of being an advanced gastric tumor, was detected on the anal side of the ulcerated lesion (Figure 1b). The biopsy specimen from the ulcerated lesion indicated a moderately or poorly differentiated tubular adenocarcinoma. Computed tomography (CT) revealed thickening of the gastric wall and findings that seemed to indicate abdominal wall invasion (Figure 1c).

**Figure 2:**
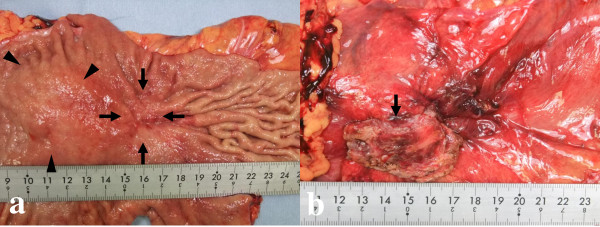
**Macroscopic examination of the specimens**. a. Upon macroscopic examination, the specimens showed an elevated and superficial depressed-type (IIa+IIc type) gastric cancer (arrow) and an elevated lesion similar to a submucosal tumor (arrow head). b. The abdominal wall (arrow) was resected together with the stomach.

**Figure 3:**
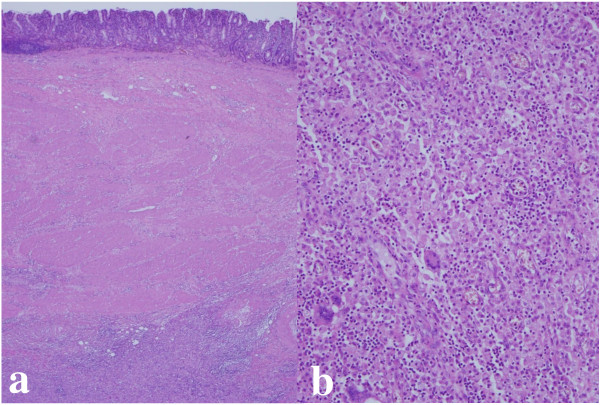
**Histopathological examination of the specimens**. Histopathological examination revealed that an SMT was located in the subserosal layer (a) and it consisted of foamy histiocytes, many lymphocytes, plasma cells, and granulocytes (b).

Open surgery was carried out and revealed that the tumor had infiltrated into the abdominal wall. Therefore, total gastrectomy and resection of the regional lymph node and parts of the abdominal wall were performed. Upon macroscopic examination, the specimens showed an elevated and superficial depressed-type (IIa+IIc type) gastric cancer, and the adjacent tumor had extended into the abdominal wall beyond the gastric serosa (Figure 2). Histopathological examination of the specimens demonstrated moderately differentiated adenocarcinoma without metastasis to the resected lymph nodes and xanthogranuloma consisting of foamy histiocytes, many lymphocytes, plasma cells, and granulocytes which were immunohistochemically positive for CD68 and were non reactive with CAM5.2, AE1/3 and S-100 protein (Figure 3). The xanthogranuloma was located near to the gastric cancer, but was not in contact with it. The patient recovered rapidly and was discharged on postoperative day 16. She has been symptom free ever since.

## Discussion

Xanthogranuloma is a tumor that is macroscopically characterized by the formation of multiple golden yellow or bright yellow nodules, and histologically, the lesion is predominantly composed of foamy histiocytes mixed with acute and chronic inflammatory cells. The pathogenesis of xanthogranuloma has not been fully established, although it is thought to be a chronic lesion associated with infection, immunological disorders, lipid transport, and lymphatic obstruction [1].

To the best of our knowledge, only seven cases of xanthogranuloma of the stomach have been reported [2-8], and the coexistence of this lesion with early gastric cancer has only been reported in 2 cases. Our histopathological inspection in these cases did not support continuity between the xanthogranuloma and early gastric cancer. Therefore, it is unclear whether early gastric cancer participates in xanthogranuloma.

Pathologically, stromal tumors such as GIST, myogenetic tumors, and neurogenic tumors account for 54 percent of all SMT, followed by heterotopic pancreas, cyst, lipoma, carcinoid, lymphangioma, and hemangioma [9]. There have been no previous cases of preoperatively diagnosed xanthogranuloma as was found in the current case.

In our case, the gastric xanthogranuloma was preoperatively misdiagnosed as an advanced gastric cancer. This occurred for the following reasons: First, a gastrointestinal endoscopic examination demonstrated an elevated lesion close to the anal side of an ulcerated lesion and a moderately or poorly differentiated adenocarcinoma was detected by the endoscopic biopsy. Second, CT indicated that the elevated lesion had invaded the abdominal wall, and a defined abdominal mass was palpable on physical examination. Therefore, the tumor was recognized as an advanced gastric cancer. Biopsy of the elevated lesion should have been carried out preoperatively to obtain a correct diagnosis in consideration of the coexistence of the two lesions.

## Conclusion

We report an extremely rare case of gastric xanthogranuloma combined with early gastric cancer. When we find SMT of the stomach, we should bear in mind not only neoplastic tumors but also inflammatory tumors. Further accumulation and investigation of gastric xanthogranuloma cases is necessary.

## Consent

Written informed consent was obtained from the patient for publication of this case report and accompanying images. A copy of the written consent is available for review by the Editor-in-Chief of this journal.

## Competing interests

The authors declare that they have no competing interests.

## Authors' contributions

HK did the literature search and writing of the manuscript. SY, YS, KA and KM collected the clinical data. RK was responsible for the histology consulting and pathology examination. All authors read and approved the final manuscript.

## References

[B1] Oberling C Retroperitoneal xanthogranuloma Am J Cancer 1935 23 477 489

[B2] Zafisaona G Inflammatory fibrous histiocytoma of the stomach. Apropos of a case of xanthogranuloma? Arch Anat Cytol Pathol 1987 35 149 153 3324981

[B3] Zhang L Huang X Li J Xanthogranuloma of the stomach: a case report Eur J Surg Oncol 1992 18 293 295 1607043

[B4] Guarino M Reale D Micoli G Tricomi P Cristofori E Xanthogranulomatous gastritis: association with xanthogranulomatous cholecystitis J Clin Pathol 1993 46 88 90 10.1136/jcp.46.1.88 8432899 PMC501124

[B5] Lespi PJ Gastric xanthogranuloma (inflammatory malignant fibrohistiocytoma). Case report and literature review Acta Gastroenterol Latinoam 1998 28 309 310 10347686

[B6] Lai HY Chen JH Chen CK Chen YF Ho YJ Yang MD Shen WC Xanthogranulomatous pseudotumor of stomach induced by perforated peptic ulcer mimicking a stromal tumor Eur Radiol 2006 16 2371 2372 10.1007/s00330-006-0188-3 16670869

[B7] Kubosawa H Yano K Oda K Shiobara M Ando K Nunomura M Sarashina H Xanthogranulomatous gastritis with pseudosarcomatous changes Pathol Int 2007 57 291 295 10.1111/j.1440-1827.2007.02089.x 17493178

[B8] Aikawa M Ishii T Nonaka K Nakao M Ishikawa K Arai S Kita H Miyazawa M Koyama I Motosugi U Ban S A case of gastric xanthogranuloma associated with early gastric cancer Nippon Shokakibyo Gakkai Zasshi 2009 106 1610 1615 19893291

[B9] Polkowski M Endoscopic ultrasound and endoscopic ultrasound-guided fine-needle biopsy for the diagnosis of malignant submucosal tumors Endoscopy 2005 37 635 645 10.1055/s-2005-861422 16010608

